# Microbial taxa related to natural hydrogen and methane emissions in serpentinite-hosted hyperalkaline springs of New Caledonia

**DOI:** 10.3389/fmicb.2023.1196516

**Published:** 2023-07-06

**Authors:** Marianne Quéméneur, Nan Mei, Christophe Monnin, Anne Postec, Sophie Guasco, Julie Jeanpert, Pierre Maurizot, Bernard Pelletier, Gaël Erauso

**Affiliations:** ^1^Aix Marseille Univ, Université de Toulon, CNRS, IRD, MIO, Marseille, France; ^2^School of Chemistry, Chemical Engineering, and Life Science, Wuhan University of Technology, Wuhan, China; ^3^Géosciences Environnement Toulouse, UMR 5563 (CNRS/UPS/IRD/CNES), Toulouse, France; ^4^Direction de l’Industrie, des Mines et de l’Energie, Nouméa, New Caledonia; ^5^Centre IRD de Nouméa, Nouméa, New Caledonia

**Keywords:** bacteria, archaea, high-pH water, microbial diversity, natural hydrogen, methane, serpentinization, geothermal springs

## Abstract

The southeastern part of New Caledonia main island (Grande Terre) is the location of a large ophiolitic formation that hosts several hyperalkaline springs discharging high pH (∼11) and warm (<40°C) fluids enriched in methane (CH_4_) and hydrogen (H_2_). These waters are produced by the serpentinization of the ultrabasic rock formations. Molecular surveys had previously revealed the prokaryotic diversity of some of these New Caledonian springs, especially from the submarine chimneys of Prony Bay hydrothermal field. Here we investigate the microbial community of hyperalkaline waters from on-land springs and their relationships with elevated concentrations of dissolved H_2_ (21.1–721.3 μmol/L) and CH_4_ (153.0–376.6 μmol/L). 16S rRNA gene analyses (metabarcoding and qPCR) provided evidence of abundant and diverse prokaryotic communities inhabiting hyperalkaline fluids at all the collected springs. The abundance of prokaryotes was positively correlated to the H_2_/CH_4_ ratio. Prokaryotes consisted mainly of bacteria that use H_2_ as an energy source, such as microaerophilic *Hydrogenophaga*/*Serpentinimonas* (detected in all sources on land) or anaerobic sulfate-reducing *Desulfonatronum*, which were exclusively found in the most reducing (E_h_ ref H_2_ ∼ -700 mV) and the most H_2_-enriched waters discharging at the intertidal spring of the Bain des Japonais. The relative abundance of a specific group of uncultured Methanosarcinales that thrive in serpentinization-driven ecosystems emitting H_2_, considered potential H_2_-consuming methanogens, was positively correlated with CH_4_ concentrations, and negatively correlated to the relative abundance of methylotrophic Gammaproteobacteria. Firmicutes were also numerous in hyperalkaline waters, and their relative abundance (e.g., *Gracilibacter* or *Dethiobacter*) was proportional to the dissolved H_2_ concentrations, but their role in the H_2_ budget remains to be assessed. The prokaryotic communities thriving in New Caledonia hyperalkaline waters are similar to those found in other serpentinite-hosted high-pH waters worldwide, such as Lost City (North Atlantic) and The Cedars (California).

## 1. Introduction

Serpentinization is a natural process of mantle rock alteration that transforms olivine into serpentine, with the parallel formation of dihydrogen (H_2_) linked to water reduction and the oxidation of metals (principally iron) contained in the minerals (mainly olivine) (Sleep et al., 2004; Klein et al., 2013; Schrenk et al., 2013; Wang et al., 2014). This H_2_ can react with carbon-bearing species such as carbonic acid to form methane (CH_4_) as well as low-molecular-weight organic compounds such as acetate and formate (Schrenk et al., 2013; Wang et al., 2014; Konn et al., 2015). These organic compounds can be sources of energy (electron donors) and of carbon for microorganisms living in deep environments (McCollom and Seewald, 2013; Ménez, 2020). The environmental characteristics of serpentinizing environments are considered similar to those that have prevailed on the early Earth and on other rocky planets like Mars, thus providing a prebiotic chemistry that may have favored the emergence of life (Nealson et al., 2005; Russell et al., 2010). Thus, the study of these ecosystems allows to address fundamental questions such as the origin and limits of life on Earth, or more applied investigations such as the search of extremophiles (i.e., alkaliphiles) for biotechnological applications or the production of natural H_2_ (also called native H_2_) (Gaucher, 2020; Truche et al., 2020).

Several ecosystems found in such serpentinization-driven environments have been found in various areas around the world: (i) underwater, such as the shallow submarine Prony Bay hydrothermal field (PBHF) located at less than 50 meters below sea level (mbsl) in the southern lagoon of New Caledonia (South Pacific) (Monnin et al., 2014; Quéméneur et al., 2014; Postec et al., 2015), the deep submarine Lost City hydrothermal field (LCHF) near the Mid-Atlantic Ridge at 700–800 mbsl (Kelley et al., 2005), and the abyssal Old City hydrothermal field at 3,100 mbsl along the southwest Indian ridge (Lecoeuvre et al., 2021), or (ii) on-land, such as The Cedars spring system (CA, USA) (Suzuki et al., 2013), the Coast Range Ophiolite (CA, USA) (Twing et al., 2017), the Samail ophiolite (Oman) (Rempfert et al., 2017) and the Voltri ophiolitic springs (Italy) (Quéméneur et al., 2015). Phylogenetically and metabolically diverse microbial communities live inside the chimneys and concretions built by the venting of anoxic, high-pH fluids (Schrenk et al., 2013). Reactions involving H_2_, CH_4_, and sulfur compounds act as the energy source for these microbial communities, indicating that serpentinization-related fluids can sustain chemosynthesis rather than photosynthesis (McCollom and Seewald, 2013).

The southern part of the New Caledonia main island (Grande Terre) is covered by a large allochthonous sheet of oceanic lithosphere (ophiolite) thrusted over continental basement at the late Eocene (Avias, 1967; Cluzel et al., 2001; Pirard et al., 2013). There, a number of high-pH springs are located either on land, in the intertidal zone of the Prony Bay or at shallow depths in this bay (Cox et al., 1982; Monnin et al., 2014, 2021; Deville and Prinzhofer, 2016; Maurizot et al., 2020). In the Prony Bay, high-pH (up to 11.2) and warm (up to 42°C) fluids enriched in H_2_ (12–30% vol of dry gas) and CH_4_ (6–14% vol of dry gas) are currently venting into the lagoon (Monnin et al., 2014; Vacquand et al., 2018). Their mixing with seawater leads to the formation of brucite-carbonate chimneys and pinnacles, reaching heights up to tens of meters, as the 38-m high Aiguille de Prony (Launay and Fontes, 1985; Quéméneur et al., 2014). Previous molecular surveys have revealed the prokaryotic diversity of the coastal submarine PBHF, with diverse aerobic and anaerobic bacteria potentially involved in H_2_ consumption (e.g., *Hydrogenophaga* and *Serpentinimonas*) or H_2_ production (e.g., Clostridiales) (Quéméneur et al., 2014; Mei et al., 2016b). In addition, a low diversity of uncultured Methanosarcinales, potentially linked to CH_4_ production or oxidation, was observed in PBHF chimneys (Quéméneur et al., 2014; Postec et al., 2015; Frouin et al., 2018), and was also found in other serpentinization-associated submarine and terrestrial sites such as The Cedars or Lost City (Kelley et al., 2005; Suzuki et al., 2013; Quéméneur et al., 2015). However, the microbial communities from on-land springs of the New Caledonia ophiolite have been only studied from the geothermal spring of La Crouen (Quéméneur et al., 2021), where bacteria potentially involved in sulfur cycle (e.g., *Candidatus* Desulfobacillus, *Thiofaba*, *Thiovirga*) and H_2_ oxidation (e.g., *Hydrogenophaga*) dominated with *Ca*. Gracilibacteria in the waters depleted in H_2_. The concentrations of H_2_ and CH_4_ in gases and waters emitted at the high-pH springs of New Caledonia are quite variable (Monnin et al., 2014, 2021; Deville and Prinzhofer, 2016) and likely to play a major role on the abundance and diversity of microorganisms.

This study used 16S rRNA gene analyses (metabarcoding and qPCR) to investigate the abundance and composition of prokaryotic communities inhabiting the high-pH fluids discharging at several on-land and intertidal springs of New Caledonia. We evaluated the relationship between the main taxa and the physicochemical characteristics of the high-pH fluids, including their contents in H_2_ and CH_4_. We also compared the dominant microbial members with those of other serpentinite-hosted hyperalkaline springs worldwide to uncover specific taxonomic bioindicators of this type of ecosystem and proxies of natural H_2_ and CH_4_ emissions.

## 2. Materials and methods

### 2.1. Study site

**FIGURE 1:**
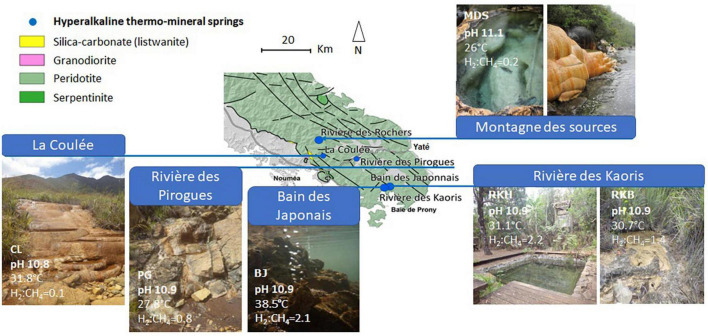
Location and photos of the high-pH springs of New Caledonia. CL corresponds to the highest pool of “La Coulée,” MDS is a pool of the site “Montagne des Sources,” PG indicates the source of the site “Rivière des Pirogues,” RKB and RKH correspond to a natural and an artificial pool of the area “Rivière des Kaoris,” respectively, and, BJ is a small intertidal vent of the site “Bain des Japonais” in the Prony Bay. The map is adapted from Maurizot et al. (2020). The H_2_:CH_4_ ratio is expressed in concentrations mol/mol.

The studied high-pH thermal springs are all located in the southeastern part of the main island (Figure 1). The water samples were collected in November–December 2014 at five different sites : (i) a shallow (10-cm deep) pool of “La Coulée” (CL) spring, close to the town of Nouméa, (ii) the source of “Rivière des Pirogues” (PG), located halfway between the cities of Nouméa and Yaté, (iii) a natural 1-m deep pool of “Montagne des Sources” (MDS), located in a natural reserve close to the city of Nouméa, (iv) a shallow (10-cm) natural pool (RKB) and the spring captured in a cemented pool 1-m deep (RKH) of “Rivière des Kaoris,” located in the Prony Bay, (v) the water venting at the “Bain des Japonais” (BJ), located in the intertidal zone of the Prony Bay (Figure 1). The spring locations have been previously described by Maurizot et al. (2020) and Monnin et al. (2021). All on-land springs are associated with carbonate deposits.

### 2.2. Sample collection

Water samples were collected in cleaned 4-L plastic bottles using sterile and pre-rinsed syringes and stored in a portable icebox until arrival at the laboratory (about 2–3 h after sampling). Duplicate samples of two liters of water were filtered through 0.2 μm pore-size Isopore polycarbonate membrane filters 47-mm (Millipore). The filters were kept at -80°C before DNA extraction. Water samples dedicated to the chemical analyses were collected in duplicate in 120-ml glass bottles sealed with butyl-rubber stoppers. At springs where gas bubbles form, free gas samples were collected in 10-ml glass vials using the water displacement technique and sealed with butyl-rubber stoppers and aluminum caps in 10-ml glass vials these samples were kept at 4°C in the Nouméa (IRD) and Marseille (MIO) laboratories. The oxidation-reduction potential (E_h_), dissolved oxygen (O_2_), pH, temperature, and conductivity were measured *in situ* using a WTW Multi 3420^®^ Multimeter with adequate probes (Monnin et al., 2021).

### 2.3. Dissolved gas analysis

Dissolved gas analysis was performed using a headspace equilibration method adapted from Magen et al. (2014). Briefly, a headspace representing 10% of the vial volume was created in the collection bottle by water displacement with argon, then the bottle was manually shaken for 1 min and placed on a shaker for 1 h. The composition of the headspace gas was determined using a Shimadzu GC 8A gas chromatograph equipped with a thermal conductivity detector (GC/TCD) and a concentric column CTR1 (Alltech, USA), as described previously (Mei et al., 2014). Argon was used as carrier gas at a flow rate of 60 mL/min; the injector and detector temperatures were fixed at 150°C. The concentrations of the dissolved gases were calculated using Henry’s law (Sander, 2015).

### 2.4. DNA extraction

The filters were transferred to a sterile 2 mL tube containing a glass bead mixture (lysing matrix E from MP Biomedicals). The bacterial and archaeal cells of the filters were disrupted by a combination of mechanical (bead beating, according MP Biomedical recommendations) and chemical lyses by addition of 1 mL of sterile bacterial lysis buffer [100 mM NaCl, 100 mM Tris pH 8.0, 50 mM EDTA, 100 μL of 10 mg/mL lysozyme (Sigma-Aldrich, St. Louis, MO, USA), 20 μL of 10 μg/mL DNase-free RNase solutions] and incubation at 37°C for 15 min. Then, 100 μL of 10% SDS, 100 μL of 10% lauryl-sarkosyl and 50 μL of proteinase K (20 mg/mL were added to the mixture and incubated at 55°C for 1 h). DNA was extracted from the lysate with 1 volume of phenol:chloroform (1:1) mixture and then with 1 volume of chloroform. Total DNA was precipitated from the aqueous phase by adding 0.7 volume of isopropanol followed by centrifugation. The DNA pellet was washed in 75% ethanol and was again collected by centrifugation. The air-dried pellet was dissolved in 30 μL of TE buffer. The DNA concentrations were measured using a Qubit^®^ fluorometer (Invitrogen).

### 2.5. Quantitative real-time PCR (qPCR)

The abundances of bacteria and archaea were determined by qPCR using, respectively, the primers set 341F/518R (Muyzer et al., 1993) and 344F/519R (Ovreås et al., 1997; Casamayor et al., 2002). The primer set used to quantify methanogens was ME3MF/ME2r’ targeting *mcrA* genes (Hales et al., 1996; Nunoura et al., 2008). The primer set used to quantify sulfate-reducers was DSRp2060f/DSR4R targeting *dsrB* genes (Wagner et al., 1998; Geets et al., 2006). The sequences of primers are given in Supplementary Table 1.

Each qPCR mixture (20 μL) contained 1X SsoAdvanced SYBR Green Supermix (Bio-Rad), 250 nM of each primer, 1 μl of DNA template (10-fold dilution series of standard PCR product or environmental DNA sample) or distilled water (negative control). All qPCR assays were performed in triplicate on a Bio-Rad CFX-96 real-time system (Bio-Rad). The qPCR cycling conditions were: 95°C for 2 min, followed by 40 cycles of a 2-step PCR protocol with a 15 sec denaturation phase at 95°C and a 15 sec annealing/elongation phase at 55°C. Fluorescence was measured at the end of each cycle. Following PCR, melt curves were generated between 65 and 95°C in 0.5°C increments, to verify PCR specificity.

qPCR standard curves were created from serial dilution of DNA standards of known concentration. For bacterial 16S rRNA and *dsrB* gene qPCR, standard DNA fragments were amplified from *Desulfovibrio vulgaris*^T^ DSM 644 using the primer sets 27F/907R (Lane, 1991) and DSR1F/DSR4R (Wagner et al., 1998), respectively. For archaeal 16S rRNA and *mcrA* gene qPCR, standard DNA fragments were amplified from *Methanosarcina barkeri*^T^ DSM 800 using the primer sets 109F/958R (Webster et al., 2006) and MLF/MLR (Luton et al., 2002), respectively. Triplicate PCR products were pooled and purified with the Nucleospin and PCR Clean-up kit (Macherey-Nagel), according to the manufacturer instructions. Purified PCR products were quantified using the BioSpec-nano Spectrophotometer (Shimadzu) and used as DNA standards. Copy number of DNA standards was calculated as described by Oldham and Duncan (2012). For each gene, the standard curve of *C*_*T*_ (threshold cycle) versus the gene copy numbers was generated by using a 10-fold dilution series from 10^8^ to 10^1^ copies per ng of DNA. For all standard curves, the coefficients of determination (R^2^) were higher than 99.0%. The precision of the assay was measured by calculating the variation in Ct values across the three replicates. The abundance of targeted genes was reported as copy numbers per L of water.

### 2.6. 16S rDNA metabarcoding analyses of microbial communities

Bacterial and archaeal 16S rRNA gene V4 hypervariable regions were amplified by PCR using the 515F/806R universal primer set (Caporaso et al., 2011), with a barcode on the forward primer, as previously described by Dowd et al. (2008), and were sequenced by the MiSeq Illumina platform of the Molecular Research Laboratory (TX, USA). Sequence data were processed using the MR DNA analysis pipeline (MR DNA, Shallowater, TX, USA). In summary, sequences were joined (overlapping pairs) and grouped by samples following the barcodes before removing them. Short sequences (<150 bp) and sequences with ambiguous base calls were removed. Remaining sequences were denoised, operational taxonomic units (OTUs) were generated, and chimeras were checked using UCHIME and removed (Edgar et al., 2011). OTUs were clustered at 97% of similarity with USEARCH (Edgar, 2010) followed by removal of singleton sequences. Finally, OTUs were taxonomically classified using BLASTn against NCBI non-redundant (NR) reference database, and the top hit was taken as a taxonomic classification. The 16S rRNA gene sequences of the dominant OTUs have been deposited in the Genbank database under the accession numbers OQ551354-OQ551410. Raw sequence data were submitted to the NCBI SRA under BioProject PRJNA974011, BioSamples SAMN35158175–SAMN35158185.

### 2.7. Statistical analyses

All statistical analyses were performed using XLSTAT 2020.5.1 (Microsoft Excel add-in program; Addinsoft, Paris, France). The alpha diversity was calculated using the Shannon (1948) and Simpson (1949) indices from OTU abundance matrix. The beta diversity was based on Bray–Curtis’s dissimilarities and a principal coordinate analysis (PCoA) (from phyla/classes) or a dendrogram (from dominant OTUs) was generated to group samples into clusters. Spearman correlations and principal component analyses (PCA) were used to evaluate the relationship between the relative abundance of microbial taxa (classes/phyla and dominant OTUs) and the physico-chemical parameters of water. *P*-values < 0.05 are statistically significant. The abundance of the dominant OTUs in the water samples was also visualized by heatmap.

## 3. Results

### 3.1. Physico-chemical parameters and dissolved gases of water samples

**FIGURE 2:**
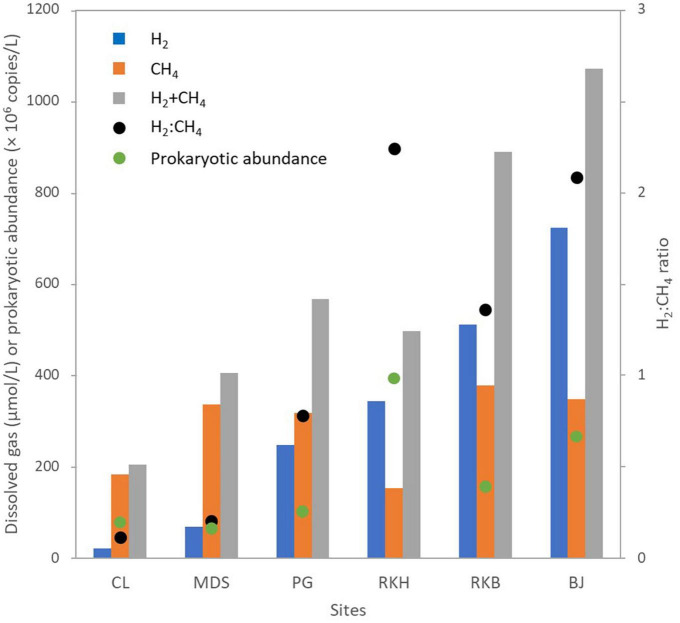
Dissolved gas (hydrogen and methane) and abundance of prokaryotes in water samples collected from the high-pH springs of New Caledonia. CL corresponds to the highest pool of “La Coulée,” MDS is a pool of the “Montagne des Sources,” PG indicates the source of the “Rivière des Pirogues,” RKB and RKH correspond to a natural and an artificial pool of the “Rivière des Kaoris,” respectively, and, BJ is a small intertidal vent of the “Bain des Japonais.” Sum and ratio of hydrogen and methane concentrations (H_2_:CH_4_ ratio) and sum of the abundance of archaea and Bacteria (total quantity of prokaryotes obtained by real-time PCR) were indicated.

The water samples collected from the six spring sites had high pH values ranging from 10.8 (CLW) to 11.1 (MDSW), and their temperatures varied from 26.0°C (MDSW) to 38.5 °C (BJW) (Table 1), as previously reported (Monnin et al., 2021). The lowest E_h_ value (-697 mV, ref. H_2_) was measured at the intertidal site of the “Bain des Japonais” (BJW). At this spring, very active gas bubbling is observed. The highest concentration of dissolved H_2_ (721.3 μmol/L) is also measured from the BJ waters (Table 1). The lowest dissolved O_2_ level (0.1 mg/L) was measured at the site of “Rivière des Kaoris” (RKB), also a site with intense gas bubbling (Monnin et al., 2014, 2021). There the concentration of dissolved CH_4_ (376.6 μmol/L) is the highest of all the studied springs, while the highest ratio of H_2_:CH_4_ was observed at the site RKH (Figure 2 and Table 1).

**TABLE 1:** *In situ* physicochemical parameters, diversity indices and gene abundances in water samples collected in the high-pH geothermal springs of New Caledonia.

Variables/samples	CLW	MDSW	PGW	RKHW	RKBW	BJW
pH*	10.8	11.1	10.9	10.9	10.9	10.9
Temperature (°C)*	31.8	26.0	27.8	31.1	30.7	38.5
O_2_ (mg/L)*	0.4	3.0	0.3	2.2	0.1	0.2
E_h_ (mV; ref. H_2_)*	324	42.0	−606.0	−176.0	−500.0	−697.0
H_2_ (μmol/L)	21.1	68.4	247.4	342.2	510.7	721.3
CH_4_ (μmol/L)	182.4	335.4	317.8	153.0	376.6	347.2
Bacterial 16S rDNA (copies/L)	3.7 × 10^7^	3.1 × 10^7^	6.3 × 10^7^	3.3 × 10^8^	1.1 × 10^8^	2.2 × 10^8^
Archaea 16S rDNA (copies/L)	8.2 × 10^6^	4.6 × 10^6^	5.1 × 10^6^	5.1 × 10^7^	2.5 × 10^7^	1.4 × 10^7^
*dsrB* (copies/L)	7.2 × 10^4^	3.0 × 10^4^	7.8 × 10^4^	5.5 × 10^4^	1.9 × 10^5^	3.5 × 10^6^
*mcrA* (copies/L)	3.9 × 10^5^	2.4 × 10^6^	3.1 × 10^6^	1.0 × 10^7^	1.7 × 10^7^	1.8 × 10^6^
Shannon index	5.29	4.84	5.25	4.58	5.23	4.10
Simpson index	0.98	0.97	0.97	0.93	0.97	0.92

*Data obtained from Monnin et al. (2021). CLW corresponds to the water collected from the highest pool of “La Coulée,” MDSW is the water collected from a pool of the site “Montagne des Sources,” PGW indicates the water of the source of the site “Rivière des Pirogues,” RKBW and RKHW correspond to a natural and an artificial pool of the area “Rivière des Kaoris,” respectively, and, BJW is the water collected from a small intertidal vent of the site “Bain des Japonais” in the Prony Bay.

### 3.2. Abundance and diversity indices of prokaryotic communities

Real-time qPCR assays and 16S rDNA metabarcoding analyses were performed on the water samples collected from the six high-pH springs. The qPCR experiments revealed that the bacterial 16S rRNA gene abundances ranged between 3.1 × 10^7^ (MDSW) and 3.3 × 10^8^ (RKHW) copies/L. The archaeal 16S rRNA gene abundances varied from 4.6 × 10^6^ (MDSW) to 5.1 × 10^7^ (RKHW) copies/L (Table 1). The abundance of bacterial 16S rRNA genes was more than one order of magnitude higher than archaeal 16S rRNA genes, as previously observed in PBHF chimneys (Quéméneur et al., 2014; Postec et al., 2015). The highest abundances of prokaryotes were measured in the waters of the PBHF sites, with maximum values in the RKH pool (displaying the highest H_2_ concentrations) (Figure 2). Prokaryotic abundances increased with increasing H_2_:CH_4_ ratio (Figure 2 and Supplementary Figure 1). The *mcrA* genes (markers of methanoarchaea) were detected in all samples but were more abundant in the waters of the Rivière des Kaoris (> 10^7^ copies/L of RKHW and RKBW). The *dsrB* genes (markers of sulfate reducers) were also detected in all samples. They were more abundant in the BJ waters (BJW), where sulfate concentrations range between 10 and 100 μM/L (Monnin et al., 2014).

The Simpson diversity indices (1-*D*) ranged between 0.92 and 0.98, while Shannon indices (*H*) varied from 4.1 to 5.2 (Table 1). Based on both indices, the lowest microbial diversity was observed in the most reducing water sample BJW (displaying the highest H_2_ concentrations), while the highest diversity was found in CLW (displaying the highest E_h_ value and the lowest H_2_ concentrations).

### 3.3. Global prokaryotic community composition

**FIGURE 3:**
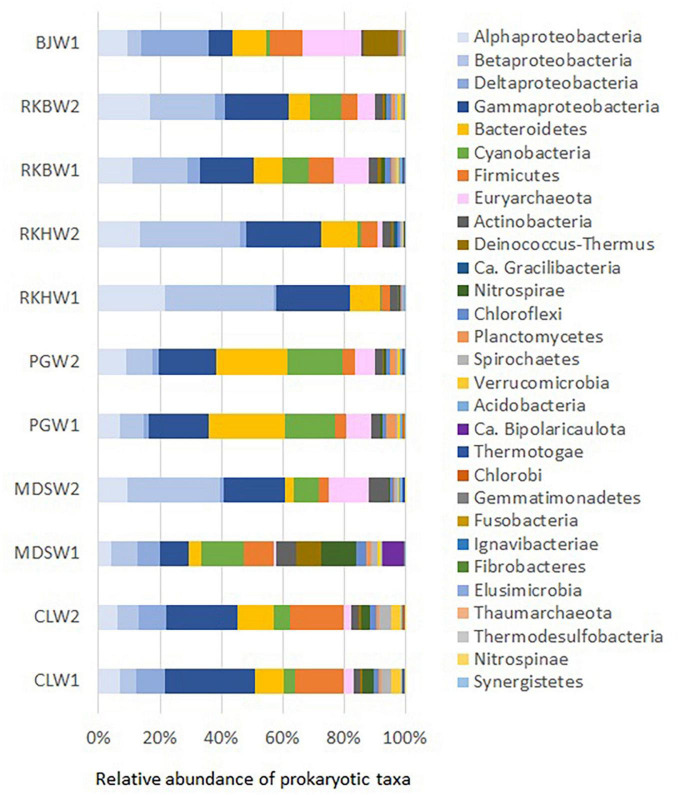
Prokaryotic phyla and proteobacterial classes (> 1% on average) in water samples collected from the high-pH springs of New Caledonia. CLW corresponds to the water of “La Coulée,” MDSW is the water of “Montagne des Sources,” PGW indicates the water of “Rivière des Pirogues,” RKBW and RKHW correspond to the waters of “Rivière des Kaoris” and BJW is the water of “Bain des Japonais.” Duplicated samples were indicated by numbers 1 or 2 (except for sample BJW).

Variation in microbial community composition was observed in the spring waters with high-pH, which were different from each other (Figure 3). This was also observed in a principal coordinates analysis (PCoA), where the replicated samples from the same spring were closely grouped (with the exception of MDS water samples), but were situated far from each other on the PCoA plot (Supplementary Figure 1).

Twenty-six different phyla were identified by 16S rRNA metabarcoding analyses of the water samples collected from the six high-pH springs (Figure 3). However, only half of these phyla (*n* = 12) were considered dominant (representing each more than 1% of prokaryotic sequences and together more than 97% of prokaryotic sequences). Proteobacteria was predominant in all samples (37.5 ± 10.0%), followed by Bacteroidetes (11.0 ± 6.9%), Firmicutes (7.9 ± 5.1%), Cyanobacteria (7.8 ± 6.1%) and Euryarchaeota (6.5 ± 5.9%; Figure 3). These five major phyla (each > 5% on average) accounted for 85% of all prokaryotic sequences. The other dominant phyla ranged between 1 and 5% of prokaryotic phyla (in average) and included Actinobacteria (3.1 ± 1.8%), Deinococcus-Thermus (2.2 ± 1.7%), Nitrospirae (2.0 ± 1.3%), Chloroflexi (1.3 ± 0.9%), Planctomycetes (1.1 ± 0.9%), Spirochaetes (1.3 ± 1.2%), Verrucomicrobia (1.1 ± 1.1%), followed by minor phyla (0.1-1%): Acidobacteria, Bipolaricaulota (formerly known as Acetothermia or OP1), Thermotogae and Gracilibacteria (formerly designated GN02/BD1-5).

The highest content of Euryarchaeota (mainly represented by Methanosarcinales) was observed in Bain des Japonais (BJ) waters (19.1%), which also displayed the highest content of Deltaproteobacteria (mainly represented by *Desulfonatronum* species). Both Deinococcus-Thermus (*Meiothermus*) and Firmicutes (*Gracilibacter*) were also detected in significant amounts in BJ waters (> 10% of prokaryotes) and in Montagne des Sources (MDS) waters (> 10%). Firmicutes were ubiquitous and abundant in all waters (> 1%) and reached a maximum in CL waters (16.7 ± 1.0%), where *Dethiobacter* species mainly represented them. The PG waters were primarily dominated by Bacteroidetes (24%) and Cyanobacteria (16%). The MDS waters displayed the highest occurrences of Bipolaricaulota.

### 3.4. Distribution and diversity of abundant prokaryotic OTUs

**FIGURE 4:**
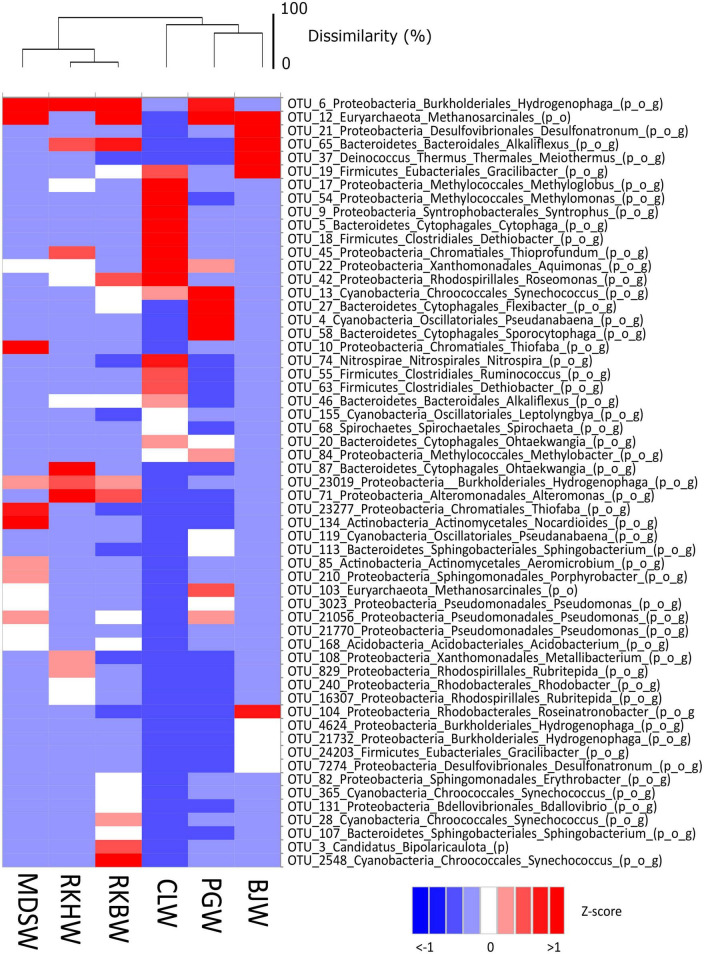
Heat map visualizing the Z-score distribution of the relative abundance of the dominant OTUs (> 1% on average) and their respective taxonomic affiliations in all water samples of the high-pH springs of New Caledonia. CLW corresponds to the water of “La Coulée,” MDSW is the water of “Montagne des Sources,” PGW indicates the water of “Rivière des Pirogues,” RKBW and RKHW correspond to the waters of “Rivière des Kaoris” and BJW is the water of “Bain des Japonais.” The average of replicates was calculated for each sampling site. The scale bar of the dendrogram represents the dissimilarity level (%) between microbial communities. The abbreviations of taxonomic ranks are p_ for phylum, o_ for order and g_ for genus.

Marked variations in the prokaryotic community of high-pH waters are also illustrated on a heatmap showing the most abundant OTUs (each > 1% on average) (Figure 4). Altogether, these 57 dominant OTUs represented almost 2/3 of the total prokaryotic sequences (60% in average).

In BJ waters (displaying the lowest E_h_ value and the highest H_2_ content), the prokaryotes were dominated by: (i) potential H_2_-oxidizing, sulfate-reducing *Desulfonatronum* OTUs (#21 and #7274, 19.5 and 1% of prokaryotes, respectively), closely related to *Desulfonatronum cooperativum* (96.7% identity; NR_043143), serpentinite-hosted PBHF clones (> 99% identity, KF886171; KT344938), and an uncultured deltaproteobacterium of deep groundwater (97.8%, LC055934), (ii) Methanosarcinales (OTU #12, 17.2% of prokaryotes) related to uncultured archaea from PBHF (KF886034 and KF886029; > 99.3% identity) and LCHF (SGYG644, SGYU755; 95.6% identity) classified as Lost City Methanosarcinales (LCMS); (iii) *Meiothermus* (OTU #37, 9.5%) closely related to *Meiothermus hypogaeus* (97.1% identity; NR_113226) and a PBHF clone (99.3% identity, KF886174), (iv) Bacteroidetes (OTU #65; 8.9%) related to uncultured bacteria from a deep subsurface gas storage aquifer (FJ168485, 90.2% identity) and deep groundwater from the Mizunami underground research laboratory (MIU) (LC055944, > 99% identity), (v) Firmicutes (OTUs #19 and #24203, 7.3 and 1.2%, respectively) affiliated with *Gracilibacter thermotolerans* (92–93%; NR_115692) and Firmicutes strains CE17 and CE8 (KX156793 and KX156784; > 95% identity) enriched from an *in situ* electrochemical experiment in a high-pH serpentinizing spring of The Cedars.

The RKH waters were dominated by: (i) H_2_-oxidizing *Hydrogenophaga* OTUs (#6 and #23019; 24.6 and 3.3% of prokaryotes) closely related to *Hydrogenophaga aquatica* (97.5% identity) and *Serpentinimonas barnesii* (NR_181590; 99.3 and 97.1% identity), (ii) *Alteromonas* OTUs (#71 and #7774; 4.8 and 1.2%), (iii) Bacteroidetes OTUs (#87 and #65, 4.6 and 2.6%) closely related to those of carbonate precipitates from the Voltri serpentinite-hosted hyperalkaline springs (99% identity; KP097469). The RKB waters, similarly to the RKH ones, were also dominated by the *Hydrogenophaga* OTUs #6 and #23019, but in smaller quantities (about half as much, 13.3 and 1.7% of prokaryotes). Both *Alteromonas* and Bacteroidetes OTUs were also less represented (∼2%), but Methanosarcinales OTUs #12 and #103 were more abundant in RKBW (6 and 0.5%), displaying higher CH_4_ content than in RKHW (∼0.5%). Bipolaricaulota OTU #3 related to *Acetothermus autotrophicum* (AP011801, 97% identity) accounted for 2.5% of RKBW prokaryotes. Cyanobacterial OTUs related to *Synechococcus* (#28, #365, and #2548) represented more than 5% of the prokaryotes.

In PG waters, the dominant OTUs were: (i) cyanobacterial *Leptolyngbya* (#4 and #13, 6.6 and 4.4% of prokaryotes) closely related to that of the mildly alkaline (pH 8.3–8.8) low-sulfur, low-carbonate Octopus hot spring (Yellowstone National Park, USA) (99% identity; KC236907, AY862014), (ii) Methanosarcinales (#12 and #103; 4.2 and 1.8%), (iii) *Hydrogenophaga* (#6; 2.7%), (iv) *Methylobacter* (#84; 1.3%).

In CL waters, the five dominant OTUs were affiliated with: (i) potential methylotrophic *Methyloglobus* (#17; 90% identity) and *Methylomonas* (#54), (ii) potential hydrocarbon-degrading *Syntrophus gentianae* (JQ346737; OTU #9; 95% identity), similar to uncultured deltaproteobacterium detected from deep groundwater (LC055948, > 98% identity), (iii) uncultured Firmicutes related to *Dethiobacter alkaliphilus* (OTUs #5, #18, #63; 94–96% identity), also detected from deep groundwater (LC055956) and from several serpentinite-hosted ecosystems (> 97% identity): PBHF (KJ149239, KF886127), Cabeço de Vide Aquifer (CVA) (AM777965), the hyperalkaline Allas Springs (Cyprus, 97.4% identity, JQ766804), the hyperalkaline spring GPS1 fed with deep groundwater at The Cedars (KC57503216S).

The MDS waters were dominated by six OTUs (representing more than 1/3 of the community) affiliated with (i) *Hydrogenophaga*/*Serpentinimonas* (#6 and #23019; 10.0 and 1.4% of prokaryotes), (ii) Methanosarcinales (#12 and #103; 10.0 and 1.1%), and (iii) sulfur-oxidizing *Thiofaba* (#10 and #23277, 8.2 and 2.4%).

### 3.5. Relationships between abundant taxa and environmental variables

**FIGURE 5:**
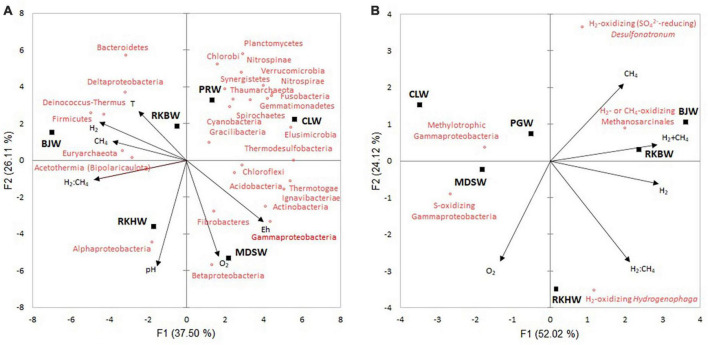
Principal component analysis (PCA) biplots show the variation among the high-pH water samples based on the physicochemical variables and the relative abundances of phyla (and proteobacterial classes) **(A)** and dominant prokaryotic functional groups **(B)**. Black squares represent water samples of high-pH springs of New Caledonia. Arrows indicate the direction of each variable’s maximum increase and strength (through the length) to the overall distribution. CLW corresponds to the water of “La Coulée,” MDSW is the water of “Montagne des Sources,” PGW indicates the water of “Rivière des Pirogues,” RKBW and RKHW correspond to the waters of “Rivière des Kaoris” and BJW is the water of “Bain des Japonais.”

Principal component analyses (PCA) was performed to identify the factors that affect the microbial community of New Caledonia high-pH spring waters (Figure 5). The first two principal components explained 63.6 and 76.1% of the data variability, for the phyla (Figure 5A) and major prokaryotic functional groups (Figure 5B), respectively. The first axis mostly separated the waters with the highest H_2_ contents (BJW, RKBW, and RKHW) from the others. The second axis separated the more oxygenated waters RKHW and MDSW from the others. Spearman’s rank correlation analyses examined the relationships between the microbial taxa, diversity indices and the environmental variables (Supplementary Tables 2–4).

At the phylum/class level, Gammaproteobacteria was positively correlated to E_h_ (*r*_S_ = 0.94, *p* < 0.05), and Deltaproteobacteria was positively correlated to temperature (*r*_S_ = 0.83, *p* < 0.05). Firmicutes were positively correlated to H_2_ (*r*_S_ = 0.96, *p* = 0.003), while the four phyla Elusimicrobia, Thermotogae, Ignavibacteria, and Thermodesulfovibrio were negatively correlated to H_2_ (*r*_s_ = −0.89 to 0.99). Euryarchaeota (including methanogens) and Bipolaricaulota (Acetothermia) were positively correlated to CH_4_ (*r*_s_ = 0.83 and 0.94, respectively; Supplementary Table 2).

Among the dominant OTUs, four OTUs (*Gracilibacter* #19, *Meiothermus* #37, Bacteroidetes #65, *Roseinatronobacter* #104) were positively correlated with H_2_. In comparison, five OTUs (Cyanobacteria #4, *Synthrophus* #9, *Dethiobacter* #63, Nitrospirae #74, *Leptolyngbya* #155) were negatively correlated with H_2_, and only one OTU (Methanosarcinales #12) was positively correlated with CH_4_ (Supplementary Table 3). Methanosarcinales (OTUs #12 and #103) were negatively correlated with methylotrophic gammaproteobacterial group (*r*_S_ = −0.93, *p* < 0.02). The sulfur-oxidizing gammaproteobacterial group was negatively correlated with H_2_ (*r*_S_ = −0.89, *p* < 0.05) (Supplementary Table 4 and Supplementary Figure 2). Prokaryotic abundances were positively correlated with H_2_:CH_4_ ratio (*r*_S_ = 0.94, *p* < 0.02). No significant correlation was observed between diversity indices and the environmental variables measured (Supplementary Table 2).

## 4. Discussion

**FIGURE 6:**
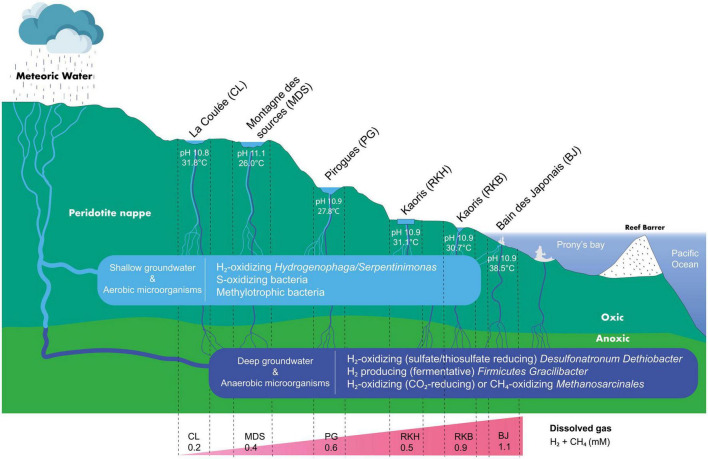
Schematic hydrothermal system diagram in southeastern New Caledonia’s high-pH springs (not to scale). The meteoric water moves underground into the subsurface, where the process of serpentinization produces hyperalkaline H_2_-rich fluids, before discharging at the geothermal springs of New Caledonia. Different microbial groups are observed depending on high-pH waters and their primary sources (shallow or deep).

The prokaryotic communities of the New Caledonian high-pH waters show several taxa previously detected in other ecosystems sustained by serpentinization, such as The Cedars and Lost City (Schrenk et al., 2013; Suzuki et al., 2013). These microbial taxa are represented by H_2_-, CH_4_-, and S-cycling prokaryotes (Figure 6), supporting the importance of oxidation or reduction of these compounds for the growth of microbial communities in New Caledonian springs.

Uncultured Methanosarcinales (OTUs #12 and #103) previously found in the submarine serpentinizing systems of PBHF (> 99% identity) (Quéméneur et al., 2014) and LCHF (> 95%) (Brazelton et al., 2010), could potentially anaerobically oxidize CH_4_ or produce it using H_2_ in an O_2_-deprived environment. Methanosarcinales phylotypes are also detected in on-land high-pH (∼11.5), H_2_- and CH_4_-rich springs of The Cedars (CA, USA) (Suzuki et al., 2013) and in the H_2_-depleted Voltri springs (Italy) (Quéméneur et al., 2015). Our study shows that the uncultured Methanosarcinales (related to LCMS phylotypes) are abundant in most New Caledonian springs but are found in small quantities (∼0.02% of the prokaryotes) in the CL waters, which have low CH_4_ and H_2_ contents. This was previously observed in the mildly alkaline (pH 9.3), CH_4_ and H_2_-poor waters of the La Crouen geothermal spring (Quéméneur et al., 2021). Moreover, the *Methanobacterium* phylotype was not detected in the hyperalkaline fluids of the studied on-land springs of New Caledonia. In contrast, this genus has been found in the alkaline geothermal spring of La Crouen (Quéméneur et al., 2021), where the novel species *Methanobacterium alkalithermotolerans* has been isolated (Mei et al., 2022), or in other on-land ecosystems sustained by serpentinization, such as the ophiolites of Samail (Oman) (Rempfert et al., 2017), Voltri (Northern Italy) (Quéméneur et al., 2015) and Zambales (Philippines) (Woycheese et al., 2015). On the other hand, in waters free from CH_4_ (e.g., La Crouen or CL), the concentrations of potential aerobic methanotrophic Gammaproteobacteria (e.g., *Methylomonas*, *Methylobacter*, *Methylophaga*) were higher (> 5%) than in the CH_4_-rich springs (<1%). These trends emphasize the importance of these potential methanogens and methanotrophs in the CH_4_ budget.

Bacterial members of the aerobic H_2_-oxidizing genera *Hydrogenophaga* (Lin et al., 2017) and *Serpentinimonas* (Bird et al., 2021) are also abundant in New Caledonian hyperalkaline springs, except in the BJ and CL waters (in which the H_2_ concentrations are contrasted, high and low, respectively). Both genera were frequently detected in waters and sediments/concretions of on-land serpentinite-hosted springs, where anoxic H_2_-rich subsurface fluids mix with oxygenated surface water (Suzuki et al., 2013; Quéméneur et al., 2015). They are present in the H_2_-rich hyperalkaline waters of The Cedars springs (Suzuki et al., 2013), as well as in the H_2_-depleted hyperalkaline waters of Voltri springs (Quéméneur et al., 2015), in the Cabeço de Vide Aquifer (Tiago and Veríssimo, 2013) and the H_2_-depleted alkaline waters of La Crouen (Quéméneur et al., 2021). The low level of dissolved H_2_ in these later spring waters could most likely be due to the high rate of H_2_ consumption by the hyperalkaliphilic H_2_-oxidizing *Serpentinimonas* (Marques et al., 2018).

BJ and CL waters displayed the lowest contents of *Hydrogenophaga*/*Serpentinimonas*, and the highest proportions of uncultured Firmicutes populations, previously identified from several serpentinizing systems. The dominant Firmicutes OTUs (#5 and #18) in CL waters were affiliated with *Dethiobacter alkaliphilus* (Sorokin et al., 2008), an anaerobic thiosulfate/polysulfide (not sulfate) reducer, polyextremophile, able to fix inorganic carbon through the Wood–Ljungdahl pathway using H_2_ as an electron donor (Melton et al., 2017). *Dethiobacter* phylotypes were abundant at Cabeço de Vide (Tiago and Veríssimo, 2013), The Cedars (Suzuki et al., 2013), the Coast Range Ophiolite (Twing et al., 2017), and the PBHF sites (Mei et al., 2016b). The dominant Firmicutes OTUs (#19 and #24203) in BJ waters are affiliated with *Gracilibacter thermotolerans* (Lee et al., 2006), a strict anaerobe able to produce H_2_ and acetate as a by-product of fermentation. *Gracilibacter* phylotypes were previously observed in the natural subsurface waters of the Coast Range Ophiolite (Twing et al., 2017) and the artificially enriched spring waters of The Cedars during an *in situ* electrochemical experiment (Rowe et al., 2017). Other potential acetogens and H_2_-producers, positively correlated with H_2_ and abundantly detected in MDS and RK waters, were affiliated with Bipolaricaulota (formerly known as Acetothermia and OP1). These microorganisms can transform low-molecular-weight organic compounds into H_2_ and acetate in the subsurface aquifer (Kadnikov et al., 2019).

The hydrogenotrophic, alkaliphilic, sulfate-reducing bacterium, *D. cooperativum*, has been isolated from a syntrophic culture growing on acetate and enriched from soda lake samples (Zhilina et al., 2005). It dominated the highly reduced waters discharged by the intertidal BJ spring (OTUs #21 and #7274), as found in a previous study (Mei et al., 2016b). These anaerobic bacteria can use sulfate, thiosulfate, elemental sulfur, or polysulfide as terminal electron acceptors. They were detected in low abundance in other on-land high-pH springs of New Caledonia (this work) and previously found in subsurface fluids of the Samail Ophiolite (Oman) (Rempfert et al., 2017). In New Caledonian high-pH waters, *Desulfonatronum* bacteria were inversely correlated with sulfur-oxidizing Gammaproteobacteria (e.g., *Thiofaba*). This points to a link between microbial activity and the sulfur chemistry, constrained by the redox conditions of the local environment. This was previously observed in borehole fluids of the Samail Ophiolite (Oman) (USA) and the California Coast Range Ophiolite (Glombitza et al., 2021).

## 5. Conclusion

The prokaryotic communities thriving in the hyperalkaline waters of several New Caledonia springs are mainly composed of microorganisms that use H_2_ as an energy source. They consist mainly of microaerophilic bacteria belonging to *Hydrogenophaga*/*Serpentinimonas* (in on-land spring waters) or anaerobic archaea belonging to a specific phylotype (designated LCMS) of uncultured Methanosarcinales (potentially able to produce CH_4_ using H_2_), which are previously detected in both submarine (i.e., Lost City, Prony Bay) (Schrenk et al., 2013; Quéméneur et al., 2014; Postec et al., 2015) and on-land serpentinizing systems (e.g., The Cedars, Voltri ophiolite) (Schrenk et al., 2013; Suzuki et al., 2013; Quéméneur et al., 2015). Thus, the relative abundance of these H_2_-consuming microorganisms could be used as signature-taxa (or taxonomic bioindicators) of serpentinite-hosted environments emitting natural H_2_.

The low abundance of *Hydrogenophaga*/*Serpentinimonas* in the water of the intertidal site BJ of the Prony Bay, where anaerobic sulfate-reducing *Desulfonatronum* proliferate together with other abundant anaerobic and thermophilic bacterial taxa (e.g., Bacteroidetes, *Meiothermus*, or *Gracilibacter*), suggests different water origins between terrestrial and marine springs, or subsurface seawater infiltrations through rock fractures (Monnin et al., 2014). Moreover, the co-existence of aerobic and anaerobic microorganisms in the hyperalkaline waters of other terrestrial hyperalkaline springs of the New Caledonia ophiolite could result in a mixing of deep (anoxic and warm) and surface (oxygenated and cold) waters before surface discharge at the various springs (Figure 6). This would corroborate a circulation pattern of the hydrothermal systems in ophiolites where waters flow through oxic and anoxic zones (Leong and Shock, 2020).

The relative abundance of uncultured Methanosarcinales-like sequences (designated LCMS), exclusively detected in serpentinite-hosted ecosystems (Frouin et al., 2018), was negatively correlated with aerobic methylobacteria, able to use CH_4_ as the sole source of carbon and energy in the waters of the hyperalkaline springs. This result suggests their implication in the consumption of CH_4_, also measured in the anoxic water generated by the serpentinization of the New Caledonia ophiolite.

Other anaerobic microbial taxa previously found in serpentinite-hosted environments were detected in New Caledonia hyperalkaline waters. It is the case of two anaerobic Firmicutes phylotypes affiliated with *Dethiobacter* and *Gracilibacter* genera and correlated with H_2_, which could also be considered as specific taxonomic bioindicators of H_2_ emissions in serpentinite-hosted environments. Although some Firmicutes bacteria have been isolated from the hyperalkaline concretions of the New Caledonian sites of the PBHF (including H_2_-producing bacteria) (Mei et al., 2014, 2016a,b; Postec et al., 2021), most remain uncultivated and their metabolisms unknown. Their role in the natural H_2_ budget remains to be assessed.

## Data availability statement

The datasets presented in this study can be found in online repositories. The names of the repository/repositories and accession number(s) can be found in this article/Supplementary material.

## Author contributions

MQ, CM, and BP sampled water and gas during the H2NAT campaign. CM performed core parameter analyses. NM performed DNA extraction and PCR assays with the help of SG. MQ processed the MiSeq Illumina sequence data and wrote the first draft of the manuscript. All authors were involved in the critical revision and approval of the final version.
