# Supplementary material for: Microbial taxa related to natural hydrogen and methane emissions in serpentinite-hosted hyperalkaline springs of New Caledonia
Source: Front Microbiol. 2023 Jul 6;14:1196516. doi: 10.3389/fmicb.2023.1196516 (PMC10359428; doi:10.3389/fmicb.2023.1196516)
Supplement: Supplementary file 1 [file Data_Sheet_1.docx]

# Supplementary Material

## **Microbial taxa related to natural hydrogen and methane emissions in serpentinite-hosted hyperalkaline springs of New Caledonia**

Marianne Quéméneur^*^, Nan Mei, Christophe Monnin, Anne Postec, Sophie Guasco, Julie Jeanpert, Pierre Maurizot, Bernard Pelletier and Gael Erauso

*Correspondence: marianne.quemeneur@ird.fr

# Figure S1. Principal Coordinate Analysis (PCoA) ordination based on Bray-Curtis distance matrix from the prokaryotic community (phyla/classes level) of water samples collected in high-pH springs of New Caledonia. The Axis 1 explains 35.0% and Axis 2 explains 16.9% of the total population variance.

#
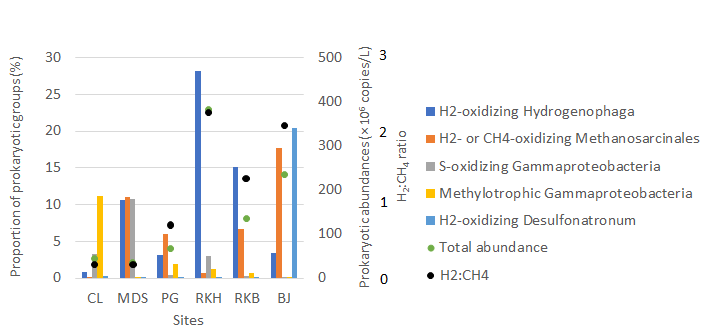


# Figure S2. Relative abundance of dominant prokaryotic groups related to dissolved hydrogen/methane ratio values and prokaryotic abundances in the high-pH waters of the New Caledonian springs.

# Table S1. Sequences of primers used in this study for the amplification of 16S rRNA genes and functional genes (*dsrAB* and *mcrA* genes).

| Primer | Sequence (5'→3') |
| --- | --- |
| Bacterial 16S rRNA genes | |
| 27F | AGA GTT TGA TCM TGG CTC AG |
| 907R | CCG TCA ATT CMT TTR AGT TT |
| 341F | CCT ACG GGA GGC AGC AG |
| 518R | ATT ACC GCG GCT GCT GG |
| Archaeal 16S rRNA genes | |
| 109F | ACK GCT CAG TAA CAC GT |
| 958R | YCC GGC GTT GAM TCC AAT T |
| 344F | ACG GGG HGC AGC AGG CGC GA |
| 519R | GWA TTA CCG CGG CKG CTG |
| Bacterial and archaeal 16S rRNA genes | |
| 515F | GTG CCA GCM GCC GCG GTA A |
| 806R | GGA CTA CHV GGG TWT CTA AT |
| *mcrA* genes | |
| MLF | GGT GGT GTM GGA TTC ACA CAR TAY GCW ACA GC |
| MLR | TTC ATT GCR TAG TTW GGR TAG TT |
| ME2R’ | TCA TBG CRT AGT TDG GRT AGT |
| ME3MF | ATG TCN GGT GGH GTM GGS TTY AC |
| ME3MF-e | ATG AGC GGT GGT GTC GGT TTC AC |
| *dsrAB* genes | |
| DSRp2060F | CAA CAT CGT YCA YAC CCA GGG |
| DSR4R | GTG TAG CAG TTA CCG CA |
| DSR1F | ACS CAC TGG AAG CAC GGC GG |

# Table S2. Spearman’s rank correlation coefficients (r) between the relative abundances of prokaryotic phyla and proteobacterial classes, as well as diversity indexes, and environmental variables of the high-pH waters of the New Caledonian springs. Values in bold correspond to significant correlations (P < 0.05). T and ORP mean temperature and oxidation-reduction potential, respectively.

| Variables | T | ORP | pH | O_2_ | H_2_ | CH_4_ |
| --- | --- | --- | --- | --- | --- | --- |
| Alphaproteobacteria | -0.029 | 0.143 | 0.377 | 0.143 | 0.371 | 0.029 |
| Betaproteobacteria | -0.429 | 0.600 | 0.464 | 0.600 | -0.257 | -0.371 |
| Deltaproteobacteria | **0.829** | -0.371 | -0.754 | -0.714 | 0.371 | 0.314 |
| Gammaproteobacteria | -0.029 | **0.943** | -0.116 | 0.600 | -0.714 | -0.657 |
| Proteobacteria | -0.200 | 0.543 | 0.464 | 0.543 | -0.086 | -0.257 |
| Fibrobacteres | -0.371 | 0.429 | 0.058 | 0.086 | -0.029 | 0.029 |
| Thermodesulfobacteria | -0.429 | 0.771 | -0.232 | 0.429 | **-0.943** | -0.257 |
| Elusimicrobia | -0.371 | 0.600 | -0.377 | 0.257 | **-0.886** | -0.314 |
| Nitrospinae | 0.257 | 0.257 | **-0.899** | -0.429 | -0.257 | -0.143 |
| Gemmatimonadetes | -0.029 | 0.486 | -0.754 | -0.200 | -0.486 | -0.200 |
| Actinobacteria | -0.486 | 0.886 | 0.058 | 0.543 | -0.714 | -0.086 |
| Planctomycetes | 0.143 | 0.086 | **-0.841** | -0.429 | -0.371 | 0.086 |
| Euryarchaeota | -0.029 | -0.486 | 0.174 | -0.314 | 0.429 | **0.829** |
| Cyanobacteria | -0.657 | -0.143 | -0.203 | -0.314 | -0.200 | 0.429 |
| Synergistetes | 0.429 | 0.143 | -0.551 | -0.029 | -0.371 | -0.657 |
| Deinococcus-Thermus | 0.771 | -0.543 | -0.377 | -0.714 | 0.829 | 0.371 |
| Gracilibacteria | 0.371 | 0.314 | -0.406 | -0.029 | -0.086 | -0.600 |
| Nitrospirae | -0.429 | 0.257 | -0.464 | -0.086 | -0.600 | -0.143 |
| Firmicutes | 0.600 | -0.771 | -0.232 | -0.771 | **0.943** | 0.429 |
| Bipolaricaulota (Acetothermia) | -0.143 | -0.600 | 0.087 | -0.600 | 0.714 | **0.943** |
| Bacteroidetes | 0.257 | -0.829 | -0.058 | -0.486 | 0.543 | -0.029 |
| Chlorobi | -0.086 | -0.200 | -0.609 | -0.543 | -0.029 | -0.029 |
| Thaumarchaeota | 0.371 | 0.371 | -0.812 | -0.314 | -0.200 | -0.257 |
| Acidobacteria | -0.771 | 0.200 | 0.087 | 0.029 | -0.314 | 0.543 |
| Ignavibacteriae | -0.657 | 0.714 | 0.058 | 0.543 | **-0.886** | -0.143 |
| Fusobacteria | -0.086 | 0.429 | -0.667 | -0.086 | -0.600 | -0.371 |
| Spirochaetes | -0.143 | 0.314 | -0.696 | -0.371 | -0.257 | 0.029 |
| Chloroflexi | -0.771 | 0.257 | -0.058 | -0.086 | -0.257 | 0.429 |
| Thermotogae | -0.371 | 0.829 | -0.058 | 0.657 | **-0.993** | -0.543 |
| Verrucomicrobia | -0.086 | 0.371 | -0.783 | -0.314 | -0.486 | 0.200 |
| Shannon index (H) | -0.257 | 0.486 | -0.507 | -0.029 | -0.714 | -0.200 |
| Simpson index (D) | -0.334 | 0.698 | -0.359 | -0.152 | -0.820 | -0.152 |

# Table S3. Spearman’s rank correlation coefficients (r) between the relative abundances of dominant prokaryotic OTUs (>1% of prokaryotic sequences) and environmental variables of the high-pH waters of the New Caledonian springs. Values in bold correspond to significant correlations (P < 0.05). T and ORP mean temperature and oxidation-reduction potential. respectively.

| Variables | T | ORP | pH | O_2_ | H_2_ | CH_4_ |
| --- | --- | --- | --- | --- | --- | --- |
| OTU_10 | **-0.993** | 0.257 | 0.493 | 0.371 | -0.371 | 0.086 |
| OTU_6 | -0.543 | 0.257 | 0.203 | 0.029 | 0.086 | 0.086 |
| OTU_23277 | **-0.993** | 0.257 | 0.493 | 0.371 | -0.371 | 0.086 |
| OTU_3023 | -0.714 | -0.257 | **0.899** | 0.543 | -0.086 | -0.086 |
| OTU_21056 | -0.714 | -0.257 | **0.899** | 0.543 | -0.086 | -0.086 |
| OTU_21770 | -0.714 | -0.257 | **0.899** | 0.543 | -0.086 | -0.086 |
| OTU_23019 | -0.543 | 0.257 | 0.203 | 0.029 | 0.086 | 0.086 |
| OTU_82 | -0.371 | -0.486 | 0.783 | 0.429 | 0.143 | -0.429 |
| OTU_85 | **-0.829** | 0.257 | 0.754 | 0.714 | -0.371 | -0.314 |
| OTU_22 | -0.143 | 0.486 | -0.696 | -0.200 | -0.600 | 0.029 |
| OTU_4624 | 0.314 | -0.600 | 0.203 | -0.486 | 0.743 | 0.600 |
| OTU_3 | 0.600 | -0.200 | 0.290 | 0.429 | **0.886** | -0.600 |
| OTU_108 | -0.314 | 0.257 | -0.551 | -0.429 | -0.143 | 0.086 |
| OTU_134 | **-0.829** | 0.257 | 0.754 | 0.714 | -0.371 | -0.314 |
| OTU_21732 | 0.143 | -0.486 | 0.261 | -0.429 | 0.829 | 0.771 |
| OTU_5 | 0.371 | 0.257 | -0.551 | 0.029 | -0.371 | -0.714 |
| OTU_13 | -0.314 | 0.257 | -0.116 | 0.371 | -0.714 | -0.714 |
| OTU_9 | -0.143 | 0.486 | -0.261 | 0.429 | **-0.829** | -0.771 |
| OTU_28 | -0.371 | 0.086 | 0.725 | 0.886 | -0.429 | -0.771 |
| OTU_37 | 0.600 | -0.714 | -0.232 | -0.771 | **0.943** | 0.429 |
| OTU_45 | 0.371 | 0.429 | -0.812 | -0.314 | -0.200 | -0.257 |
| OTU_71 | -0.486 | -0.029 | 0.348 | -0.086 | 0.371 | 0.257 |
| OTU_19 | 0.829 | -0.029 | -0.116 | 0.086 | **0.843** | -0.743 |
| OTU_17 | 0.086 | 0.657 | -0.667 | -0.086 | -0.429 | -0.314 |
| OTU_18 | 0.429 | -0.029 | -0.406 | 0.086 | -0.629 | -0.543 |
| OTU_210 | **-0.886** | 0.086 | 0.551 | 0.143 | 0.029 | 0.429 |
| OTU_4 | -0.486 | 0.371 | -0.058 | 0.429 | **-0.829** | -0.543 |
| OTU_46 | 0.314 | 0.714 | -0.348 | 0.257 | -0.371 | -0.429 |
| OTU_21 | 0.600 | -0.714 | 0.203 | 0.029 | 0.371 | -0.371 |
| OTU_20 | -0.086 | 0.486 | -0.667 | -0.086 | -0.600 | -0.371 |
| OTU_55 | 0.086 | 0.771 | -0.348 | 0.314 | -0.543 | -0.543 |
| OTU_42 | 0.086 | 0.771 | -0.348 | 0.314 | -0.543 | -0.543 |
| OTU_27 | -0.600 | -0.143 | 0.232 | 0.086 | -0.086 | -0.314 |
| OTU_84 | -0.200 | 0.200 | -0.348 | 0.086 | -0.543 | -0.600 |
| OTU_12 | 0.029 | -0.600 | 0.754 | 0.200 | 0.786 | **0.812** |
| OTU_54 | -0.029 | 0.600 | -0.754 | -0.200 | -0.486 | -0.200 |
| OTU_155 | -0.143 | 0.429 | -0.116 | 0.543 | **-0.886** | -0.657 |
| OTU_58 | -0.714 | -0.086 | 0.464 | 0.371 | -0.257 | -0.429 |
| OTU_65 | 0.600 | -0.714 | -0.232 | -0.771 | **0.943** | 0.429 |
| OTU_2548 | 0.029 | -0.257 | -0.116 | -0.143 | 0.143 | -0.486 |
| OTU_63 | -0.200 | 0.600 | 0.029 | 0.714 | **-0.943** | -0.600 |
| OTU_119 | -0.314 | 0.257 | -0.116 | 0.371 | -0.714 | -0.714 |
| OTU_104 | 0.314 | -0.600 | 0.203 | -0.486 | **0.943** | 0.600 |
| OTU_87 | -0.257 | 0.143 | -0.232 | -0.257 | 0.086 | -0.086 |
| OTU_240 | -0.143 | -0.429 | 0.058 | -0.486 | 0.657 | 0.257 |
| OTU_107 | -0.314 | 0.600 | 0.406 | 0.771 | -0.486 | -0.600 |
| OTU_68 | 0.086 | 0.486 | -0.406 | 0.257 | -0.600 | -0.771 |
| OTU_74 | -0.143 | 0.486 | -0.261 | 0.429 | **-0.829** | -0.771 |
| OTU_113 | -0.771 | -0.257 | 0.261 | -0.200 | 0.143 | 0.314 |
| OTU_365 | 0.143 | -0.143 | -0.174 | 0.029 | -0.086 | -0.714 |
| OTU_131 | -0.486 | 0.200 | 0.377 | 0.257 | 0.029 | -0.200 |
| OTU_829 | -0.543 | 0.257 | 0.203 | 0.029 | 0.086 | 0.086 |
| OTU_168 | -0.600 | -0.200 | **0.986** | 0.657 | -0.029 | -0.200 |
| OTU_24203 | 0.886 | -0.257 | -0.116 | -0.143 | 0.486 | -0.086 |
| OTU_16307 | -0.543 | 0.257 | 0.203 | 0.029 | 0.086 | 0.086 |
| OTU_103 | -0.657 | -0.429 | 0.754 | 0.371 | 0.629 | 0.643 |
| OTU_7274 | 0.829 | -0.314 | -0.174 | 0.029 | 0.086 | -0.371 |

# Table S4. Spearman’s rank correlation coefficients (r) between the dissolved hydrogen and methane values and the relative abundances of associated prokaryotic groups in the high-pH waters of the New Caledonian springs. Values in bold correspond to significant correlations (P < 0.05).

| Variables | H_2_ | CH_4_ | H_2_+CH_4_ | H_2_:CH_4_ | 1-  H_2_-ox bacteria | 2-  H_2_- or CH_4_-ox archaea | 3-  S-ox bacteria | 4- Methylo bacteria | 5- Anaer. H_2_-ox bacteria | 6- Total  prok. abund. |
| --- | --- | --- | --- | --- | --- | --- | --- | --- | --- | --- |
| 1- H_2_-ox bacteria (*Hydrogenophaga*) | 0.486 | 0.086 | 0.257 | 0.714 | **1** | 0.200 | -0.086 | -0.464 | -0.696 | 0.543 |
| 2- H_2_- or CH_4_-ox archaea (Methanosarcinales) | 0.600 | 0.771 | 0.657 | 0.257 | 0.200 | **1** | -0.429 | **-0.928** | 0.232 | 0.029 |
| 3- S-ox bacteria (Gammaproteobacteria) | **-0.886** | -0.543 | **-0.943** | -0.600 | -0.086 | -0.429 | **1** | 0.232 | -0.522 | -0.657 |
| 4- Methylotrophic bacteria (Gammaproteobacteria) | -0.551 | -0.638 | -0.493 | -0.348 | -0.464 | **-0.928** | 0.232 | **1** | 0.029 | -0.087 |
| 5- H_2_-oxidizing anaerobes *Desulfonatronum* | 0.203 | 0.406 | 0.348 | -0.232 | -0.696 | 0.232 | -0.522 | 0.029 | **1** | -0.058 |
| 6- Total prokaryotic abundance | 0.771 | -0.086 | 0.600 | **0.943** | 0.543 | 0.029 | -0.657 | -0.087 | -0.058 | **1** |
